# Supplementary material for: High quality Bathyarchaeia MAGs from lignocellulose-impacted environments elucidate metabolism and evolutionary mechanisms
Source: ISME Commun. 2024 Dec 10;4(1):ycae156. doi: 10.1093/ismeco/ycae156 (PMC11697101; doi:10.1093/ismeco/ycae156)
Supplement: Supplementary_data_ycae156 [file supplementary_data_ycae156.zip › Content of Supplementary files_revised.docx]

Supplementary text and figures: Supplementary Text S1 – S9 and Supplementary Figure S1 – S12

Supplementary Table S1 Summaries of Metagenomic Data:

Table S1A Summary data and quality statistics for the MAG constructed in this study.

Table S1B Amplicon data from the mills samples used for metagenome sequencing.

Table S1C Summary of the metagenomes used for constructing Bathyarchaeia MAGs.

Table S1D Representative Bathyarchaeia MAGs from the genome taxonomy database included in the phylogenomic analysis.

Table S1E Publicly available MAGs included in the pangenome analysis.

Table S1F Amplicon data from the cultures investigated.

Supplementary Table S2: ANI between MAGs.

Supplementary Table S3: Annotation data for Bathy6-F90_cMAG_MillC.

Supplementary Table S4: Pangenome data and proteomic data.
